# Cultural and structural barriers to HPV vaccine acceptance among young women in the United Arab Emirates

**DOI:** 10.1371/journal.pone.0346507

**Published:** 2026-10-30

**Authors:** Ream Langhe, Alaa AlKhatib, Aya Saber, Dana Saqr, Raniya Al-Janabi, Sarah Ahmed, Teresita Kelly, Ibrahim Mahmoud, Hiba Jawdat Barqawi

**Affiliations:** 1 Department of Clinical Sciences, College of Medicine, University of Sharjah, Sharjah, United Arab Emirates; 2 Faculty of Medicine, University College Dublin, Dublin, Ireland; 3 Department of Family Medicine and Behavioural Sciences, University of Sharjah, Sharjah, United Arab Emirates; Regional Health Care and Social Agency of Lodi, ITALY

## Abstract

**Background:**

Human papillomavirus (HPV) is a common sexually transmitted virus, and oncogenic genotypes are linked to cervical cancer. Vaccination reduces cervical cancer burden, yet uptake remains suboptimal in the United Arab Emirates (UAE) despite national immunisation initiatives. This exploratory study aimed to identify and characterize potential cultural and structural barriers that may impede vaccine uptake among educated, digitally connected women in the UAE to inform future, more representative research.

**Methods:**

A cross-sectional, descriptive study surveyed 1455 women aged 18–30 years across all emirates regarding vaccination status, cultural beliefs, and institutional factors influencing vaccine acceptance and uptake.

**Results:**

The participants were predominantly educated, non-Emirati, digitally connected young women (mean age 22.4 years, 77.7% non-Emirati, 44.8% healthcare-affiliated). While 58.8% respondents viewed vaccination as culturally acceptable, only 26.2% admitted to being vaccinated. The most commonly reported barriers to vaccination included concerns about side effects, feeling too young for vaccination, and fear of family judgment (47.5%, 28.4%, 27.8%, respectively). The most reported reason for receiving the vaccine was its inclusion in the immunisation schedule (37.9%), followed by parental consent and encouragement (36.6%), and for self-protection purposes (25.6%). Furthermore, acceptance increased when the vaccine was presented as a cancer prevention strategy (64.6%), offered at more convenient location (63.8%) or made more affordable (61.5%).

**Conclusion:**

Overall, the primary barriers to vaccination included concerns regarding the fear of public judgment, reflecting a combination of informational gaps, timing-related considerations, and sociocultural influences affecting HPV vaccine uptake. Future research employing representative sampling is needed to determine whether these barriers generalize to broader UAE populations including non-digitally connected, non-healthcare workers, and Emirati women. Once population level barriers are identified through representative research, public health strategies can be designed to address context-appropriate concerns through targeted education on vaccine safety and culturally sensitive engagement with families and young women to improve uptake.

## Introduction

Human papillomavirus (HPV) is one of the most common sexually transmitted viruses, with over 200 genotypes [1]. Among these, at least 14 are classified as high-risk due to their oncogenic properties, particularly types 16 and 18 [2]. Persistent infection with high-risk types can lead to cervical intraepithelial neoplasia, which, if left untreated, may progress to cervical cancer [3].

According to the World Health Organisation (WHO), cervical cancer is the fourth most common cancer among women globally, with over 660,000 cases and 350,000 deaths reported in 2022 [1]. In 2020, the WHO launched a global strategy to vaccinate 90% of girls by 15 years of age worldwide against HPV by 2030 [4]. Despite these efforts, global vaccination uptake remains suboptimal, perpetuating the incidence of preventable gynecologic pathologies. In 2023, first-dose coverage among girls aged 9–14 years reached only 27%, underscoring persistent gaps in implementation and acceptance [5].

The United Arab Emirates (UAE) was the first country in the Middle East and North Africa (MENA) region to introduce HPV vaccination as part of a public healthcare initiative. In 2008, Abu Dhabi launched a school-based HPV vaccination program, offering the vaccine free of charge to girls aged 15–17 years [6]. Other Emirates were slower to adopt these immunisation strategies, and the timeline for implementing the vaccine in each Emirate is not publicly available. By 2018, the UAE became the first country in the region to integrate the HPV vaccine into its national immunisation programmes, targeting schoolgirls aged 13–14 [6].

The Ministry of Health and Prevention (MoHAP) launched a national strategy that aims to vaccinate 90% of girls before the age of 15 years against HPV by the year 2030. Furthermore, the UAE expanded the programme to include boys aged 13–14 [7]. Despite having the highest documented rates of HPV vaccination in the Gulf Cooperation Council (GCC) in 2024, the uptake of the vaccine remained low, at around 18.9% among women aged 18–39 [8]. The low coverage rate suggests the presence of persistent barriers that hinder the success of national immunisation strategies. These barriers are likely multifactorial, including cultural stigma, misconceptions about vaccine safety, and reluctance to discuss sexually transmitted infections.

Comparable challenges have been observed across the GCC region, with studies from Saudi Arabia reporting that poor knowledge and awareness of HPV and its vaccine, among both the general population and university students [9, 10]. In Qatar, a study identified parental concerns about vaccine safety, perceived commercial motives, and discomfort discussing sexual health with adolescents as key barriers to HPV vaccine acceptance [11]. Social, cultural, and religious factors may further reinforce stigma around a vaccine, often misattributed to sexual activity. Addressing these barriers through targeted strategies is essential for improving vaccine uptake and reducing HPV-related gynaecologic disease.

This study aims to address gaps in the literature by assessing barriers and factors influencing HPV vaccine acceptance and uptake in the UAE. By analysing the potential obstacles in a culturally and religiously diverse context, the findings will inform gynaecologic prevention strategies and guide targeted interventions to boost vaccination coverage. Strengthening HPV vaccine uptake is essential in reducing the burden of HPV‑related diseases in the UAE.

## Methodology

### Study design and target population

A cross-sectional, descriptive study was conducted from 10 June to 31 August 2025 to assess the prevalence of HPV vaccination among women aged 18–30 years in the UAE and the potential barriers to vaccination.

Inclusion criteria: women aged 18–30, residing in the UAE, including UAE nationals and non-nationals who were fluent in English or Arabic. Exclusion criteria included participants outside of the eligible age range, individuals who failed to complete all mandatory questionnaire items (incomplete questionnaires), those who provided inconsistent responses, such as contradictory answers across related questions, and non-resident visitors or tourists.

Ethical approval was obtained from the Research Ethics Committee of the University of Sharjah in compliance with ethical guidelines for human research (REC-25-03-18-03-F).

### Sampling strategy

Convenience sampling was used to recruit participants. Participants were approached in public venues across all emirates in the UAE, with a focus on the three largest emirates (Abu Dhabi, Sharjah and Dubai), as they constitute the majority of the UAE’s population. An electronic link to the questionnaire was distributed to the target population via social media platforms (including WhatsApp, Instagram, X) and the university mailing list.

### Questionnaire development and validation

A structured self-administered questionnaire was developed based on validated tools and a literature review. Items assessing knowledge, barriers, attitudes and practices regarding HPV vaccination were adapted from published validated questionnaires [12 - 14] and modified to fit the study objectives and target population. The questionnaire was initially developed in English and was later translated into Arabic to ensure cultural relevance.

The final questionnaire included 17 items across five domains: demographics, HPV vaccine knowledge and uptake, cultural and structural barriers, attitudes toward HPV vaccination and vaccination practices. Both the English and Arabic versions were piloted with 10 randomly selected participants to assess the linguistic clarity and cultural relevance of the questionnaire and the responses from the pilot studies were excluded from the final data analysis.

### Sample size calculation

The minimum required sample size for the study was calculated using the standard formula for cross-sectional studies. Using a 95% confidence level, 5% margin of error, and 50% expected prevalence, the minimum sample size was 385. To enhance statistical power, 1000 participants were targeted. A total of 1468 valid responses were obtained and analysed, which exceeded the minimum required sample size and contributed to the reliability of the results.

### Data collection

Data were collected electronically using Google Forms. An information sheet was provided at the beginning of the questionnaire, and electronic informed consent was obtained from participants who proceeded to the survey after reading it. No personal identifiable information was collected. The dataset was exported to Microsoft Excel for secure storage and analysis, with access restricted to the research team. Before analysis, the dataset was cleaned by screening for and removing incomplete submissions, missing data and responses containing logical inconsistencies or contradictions.

### Scoring and interpreting HPV cultural barriers scale

The HPV Cultural Barriers Scale was scored using a five-point Likert scale ranging from 1 (strongly agree) to 5 (strongly disagree). Items 4, 5, and 9–13 were positively worded statements reflecting cultural acceptance of HPV vaccination and were therefore reverse-coded so that higher scores consistently represented lower levels of cultural barriers. After reverse coding, total scores reflected attitudes toward HPV vaccination, with lower scores indicating stronger cultural resistance and higher scores indicating greater cultural acceptance.

### Statistical analysis

Descriptive statistics were used to describe participants’ characteristics. For continuous variables, the mean and standard deviation (SD) were presented, while categorical variables were presented frequencies and percentages. To compare mean HPV Cultural Barriers Scale scores across participant characteristics, independent t-tests and one-way ANOVA were performed.

Multiple linear regression analysis was conducted to identify factors associated with cultural barriers toward HPV vaccine uptake. Variables entered into the model included age group, nationality, healthcare background, Emirate of residence, and HPV vaccination status. The Cultural Barriers Scale was analyzed among all participants according to their self-reported vaccination status categories, including vaccinated, unvaccinated, and those uncertain of their vaccination status. Participants who reported not knowing their vaccination status were retained in the analysis as a separate category to avoid unnecessary exclusion of responses and to better reflect the overall awareness and uncertainty surrounding HPV vaccination uptake within the study population. Statistical significance was set at p < 0.05. Data analysis was performed using IBM SPSS (Version 28.0, IBM Corp, Armonk, NY, USA).

## Results

### Participant characteristics

Table 1 presents the demographic characteristics of the participants. A total of 1,455 women participated (mean age 22.4 ± 3.6 years). Most participants were aged 18–24 years (76.4%), single (88.2%), and non-Emirati (77.7%). Nearly half of the participants (44.8%) were healthcare students or workers.

**Table 1:** Participants characteristics, N = 1455.

Variable	n (%)
**Age, years**	
18-24	1111 (76.4)
25-30	344 (23.6)
**Nationality**	
Emirati	324 (22.3)
Non-Emirati	1131 (77.7)
**Marital status**	
Single	1284 (88.2)
Married	171 (11.8)
**Emirate**	
Abu Dhabi	235 (16.2)
Ajman	143 (9.8)
Dubai	377 (25.9)
Sharjah	623 (42.8)
RAK/UAQ/Fujairah	77 (5.3)
**Healthcare student/worker**	
Yes	652 (44.8)
No	803 (55.2)

RAK = Ras Al Khaimah; UAQ = Umm Al Quwain.

### HPV vaccination knowledge and uptake

Table 2 shows that having received the HPV vaccine, 35.3% reported not receiving the vaccine, and 38.4% were unsure of their vaccination status. Participants who were uncertain of their vaccination status represented the largest response group. This finding complicates the interpretation of the vaccination rate within the study population. Knowledge of vaccine specifics was also limited: only 36.8% correctly identified the recommended vaccination age (13–17 years), and 59.2% correctly recognized that both sexes should be vaccinated.

**Table 2:** HPV vaccination knowledge, attitudes, and access among participants, N = 1455.

Variable	n (%)
**HPV vaccination status**	
Yes	383 (26.2)
No	514 (35.3)
I do not know	558 (38.4)
**Emirate of HPV vaccination**	
Abu Dhabi	123 (32.1)
Ajman	15 (3.9)
Dubai	64 (16.7)
Sharjah	101 (26.4)
RAK/UAQ/Fujairah	20 (5.2)
Outside UAE	60 (15.7)
**Recommended HPV vaccination age in UAE**	36 (5.7)
At birth	111 (7.6)
1 - 12	235 (16.2)
13-17	535 (36.8)
18-45	274 (18.8)
> 45	7 (0.5)
I do not know	293 (20.1)
**Who should take vaccination**	
Males	10 (0.7)
Females	328 (22.5)
Both	862 (59.2)
I do not know	255 (17.5)
**Doctor-advised about HPV**	
Yes	336 (23.1)
No	1119 (76.9)
**School/university teaches HPV**	
Yes	504 (34.6)
No	951 (65.4)
**HPV vaccine availability at local clinic/hospital**	
Yes	369 (25.4)
No	38 (2.6)
I do not know	1048 (72.0)
**Recommend HPV vaccine to friends and family**	
Yes	915 (62.9)
No	57 (3.9)
I do not know	483 (33.2)
**Doctor advice reliability**	
Yes	1101 (75.7)
No	32 (2.2)
I do not know	322 (22.1)
**Girls vaccinated before first sexual activity**	
Yes	1028 (70.6)
No	39 (2.7)
I do not know	388 (26.7)

SD = Standard deviation; RAK = Ras Al Khaimah; UAQ = Umm Al Quwain.

Healthcare engagement was limited, with only 23.1% of participants reporting receiving advice from a physician, and 34.6% reporting receiving education about vaccination through school or university. Awareness of local vaccine availability was low, with only 25.4% of participants reporting knowledge of vaccine availability, while 72% were unsure whether the vaccine was offered at their local clinic or hospital. Despite this, trust in healthcare professionals was high with 75.7% of participants considering doctors’ advice on HPV vaccination reliable, 70.6% supporting vaccinating girls before their first sexual intercourse, and 62.9% reporting that they would recommend the vaccine to others.

### Cultural barriers

Table 3 shows responses to 16 statements assessing cultural barriers to HPV vaccination among non-vaccinated women. The main reported barriers were concerns about side effects (47.5%), feeling too young for vaccination (28.4%), and fear of family judgment (27.8%) as shown in Fig 1. Embarrassment discussing the vaccine (27.0%), hearing negative opinions about the vaccine (26.1%), believing that only people in intimate relationships need vaccination (25.5%),and perceiving the topic as culturally taboo (20.6%) were also notable.

**Table 3:** Cultural barriers among non-vaccinated women, N = 514.

Item	Agree/Strongly agree	Neutral	Disagree/Strongly disagree
I hesitate to ask because of embarrassment	139 (27.0)	127 (26.7)	238 (46.3)
I would feel judged by family if I took the vaccine.	143 (27.8)	106 (20.6)	265 (51.6)
Avoided learning about the vaccine because it is taboo.	106 (20.6)	112 (21.8)	296 (57.6)
Culturally acceptable to get the vaccine.	302 (58.8)	142 (27.6)	70 (13.6)
Getting the vaccine before marriage is culturally acceptable.	279 (54.3)	159 (38.9)	76 (14.8)
Heard negative opinions about the HPV vaccine.	134 (26.1)	192 (37.4)	188 (36.6)
I’m too young for vaccine.	146 (28.4)	170 (33.1)	198 (38.5)
Religious beliefs affect my decision to get the vaccine.	93 (18.1)	149 (29.0)	272 (52.9)
I know where to access the vaccine.	157 (30.5)	142 (27.6)	215 (41.8)
I’m likely to take the vaccine if it was offered in my university/workplace.	328 (63.8)	132 (25.7)	54 (10.5)
I am more likely to take the vaccine if it was free/cheaper.	316 (61.5)	142 (27.6)	56 (10.9)
I believe the HPV vaccine should be given as a compulsory school academic requirement.	297 (57.8)	158 (30.7)	59 (11.5)
I would be more willing to take the HPV vaccine if it was promoted as a cancer-prevention vaccine rather than an STI-related vaccine.	332 (64.6)	123 (26.8)	44 (8.6)
I worry about the side effects of the HPV vaccine	244 (47.5)	172 (33.5)	98 (19.1)
Only people engaged in intimate relations need the HPV vaccine	131 (25.5)	164 (31.9)	219 (42.6)
My family does not allow me to take the vaccine.	85 (16.5)	165 (32.1)	264 (51.4)

**Fig 1:**
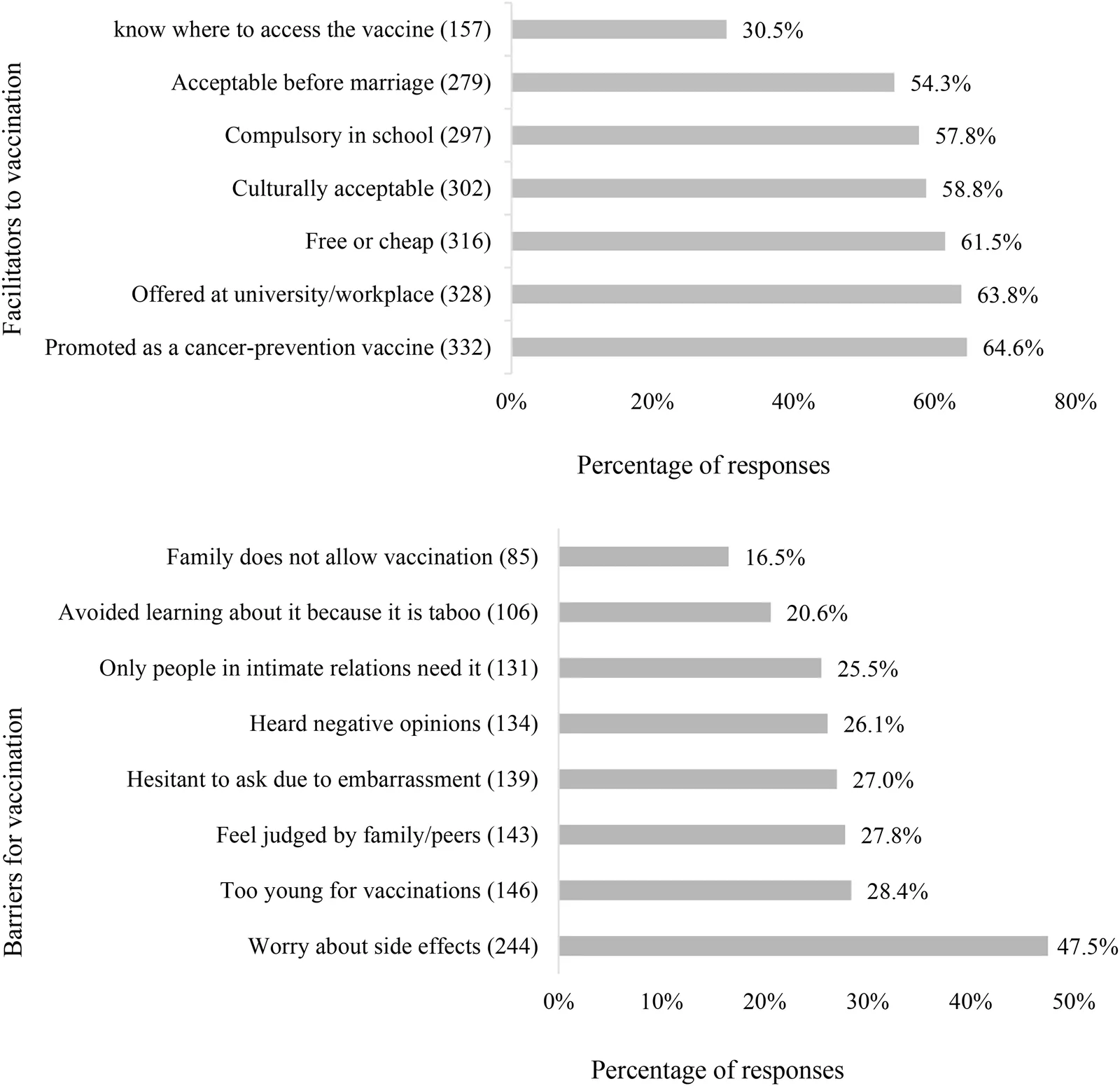
Common facilitators and barriers to HPV vaccination among non-vaccinated women (n = 514). **(A)** Facilitators for HPV vaccination. **(B)** Barriers to HPV vaccination.

58.8% of participants viewed HPV vaccination as culturally acceptable, and 54.3% considered it acceptable before marriage. Other facilitators included availability of the vaccine at the workplace or university (63.8%), reduced cost (61.5%), framing vaccination as cancer prevention rather than STI prevention (64.6%), and support for making the vaccine a compulsory school requirement (57.8%). However, only 30.5% of participants reported knowing where to access the HPV vaccine, indicating a substantial gap in awareness of vaccine availability despite these otherwise favorable attitudes (Fig 1).

### Cultural barriers scale analysis

Table 4 shows the HPV cultural carriers Scale scores by participant characteristics. Older participants, non-Emiratis, those residing in Abu Dhabi, healthcare students/workers, and vaccinated participants demonstrated greater cultural acceptance, (p < 0.05).

**Table 4:** HPV cultural barriers scale scores by participant characteristics.

Variable	Mean (SD)	P value
**Age, years**		**0.002**
18 − 24	55.7 (9.2)	
25 − 30	57.6 (9.5)	
**Nationality**		**0.008**
Emirati	55.0 (9.5)	
Non-Emirati	56.5 (9.3)	
**Marital status**		0.706
Single	56.1 (9.4)	
Married	56.4 (8.7)	
**Emirate**		**< 0.001**
Abu Dhabi	59.3 (9.6)	
Ajman	54.9 (8.4)	
Dubai	56.2 (9.2)	
Sharjah	55.3 (9.3)	
RAK/UAQ/Fujairah	55.5 (9.4)	
**Healthcare student/worker**		**< 0.001**
Yes	58 (10.0)	
No	54.7 (8.5)	
**HPV vaccination status**		**< 0.001**
Yes	60.4 (10.3)	
No	54.7 (8.7)	
Do not know	54.7 (8.2)	

P values in bold are statistically significant; SD = Standard deviation; RAK = Ras Al Khaimah; UAQ = Umm Al Quwain.

### Factors associated with HPV cultural barriers scale scores (multivariable analysis)

In the adjusted multiple linear regression model, higher HPV Cultural Barriers Scale scores were independently associated with older age (B = 1.44, p = 0.010) and non-Emirati nationality (B = 1.40, p = 0.013). Lower scores were observed among women who were not healthcare students/workers (B = −2.45, p < 0.001), women from Sharjah (B = −1.68, p = 0.018) and Ajman (B = −2.23, p = 0.020), and those who were not vaccinated (B = −5.15, p < 0.001) or were unsure of their vaccination status (B = −4.70, p < 0.001), compared with their respective reference groups (Table 5).

**Table 5:** Multiple linear regression analysis of factors associated with HPV Cultural Barriers Scale scores (N = 1,455).

Variable	B	SE	Standardized β	95% CI	P value
Age (25 − 30 vs 18 − 24)	1.44	0.56	0.066	0.34 to 2.54	**0.010**
Nationality (Non-Emirati vs Emirati)	1.40	0.57	0.063	0.29 to 2.52	**0.013**
Healthcare student/worker (No vs Yes)	−2.45	0.48	−0.130	−3.38 to −1.51	**< 0.001**
Dubai vs Abu Dhabi	−1.15	0.76	−0.054	−2.63 to 0.34	0.130
Sharjah vs Abu Dhabi	−1.68	0.71	−0.089	−3.07 to −0.28	**0.018**
Ajman vs Abu Dhabi	−2.23	0.96	−0.071	−4.11 to −0.35	**0.020**
RAK/UAQ/Fujairah vs Abu Dhabi	−1.98	1.18	−0.047	−4.28 to 0.33	0.093
Not vaccinated vs vaccinated	−5.15	0.62	−0.264	−6.37 to −3.93	**< 0.001**
Do not know vs vaccinated	−4.70	0.62	−0.245	−5.90 to −3.49	**< 0.001**

P values in bold are statistically significant; B = unstandardized regression coefficient; SE = standard error; CI = confidence interval; CI = Confidence interval; RAK = Ras Al Khaimah; UAQ = Umm Al Quwain.

### Reasons for Vaccination

The most frequently reported reasons for receiving the HPV vaccine were inclusion in the national **vaccination schedule (37.9%),** followed by **parental consent (36.6%)**, and **self-protection (25.6%) (**Fig 2**).** These findings indicate that external recommendations and parental influence played a stronger role in vaccination decisions than personal motivation.

**Fig 2:**
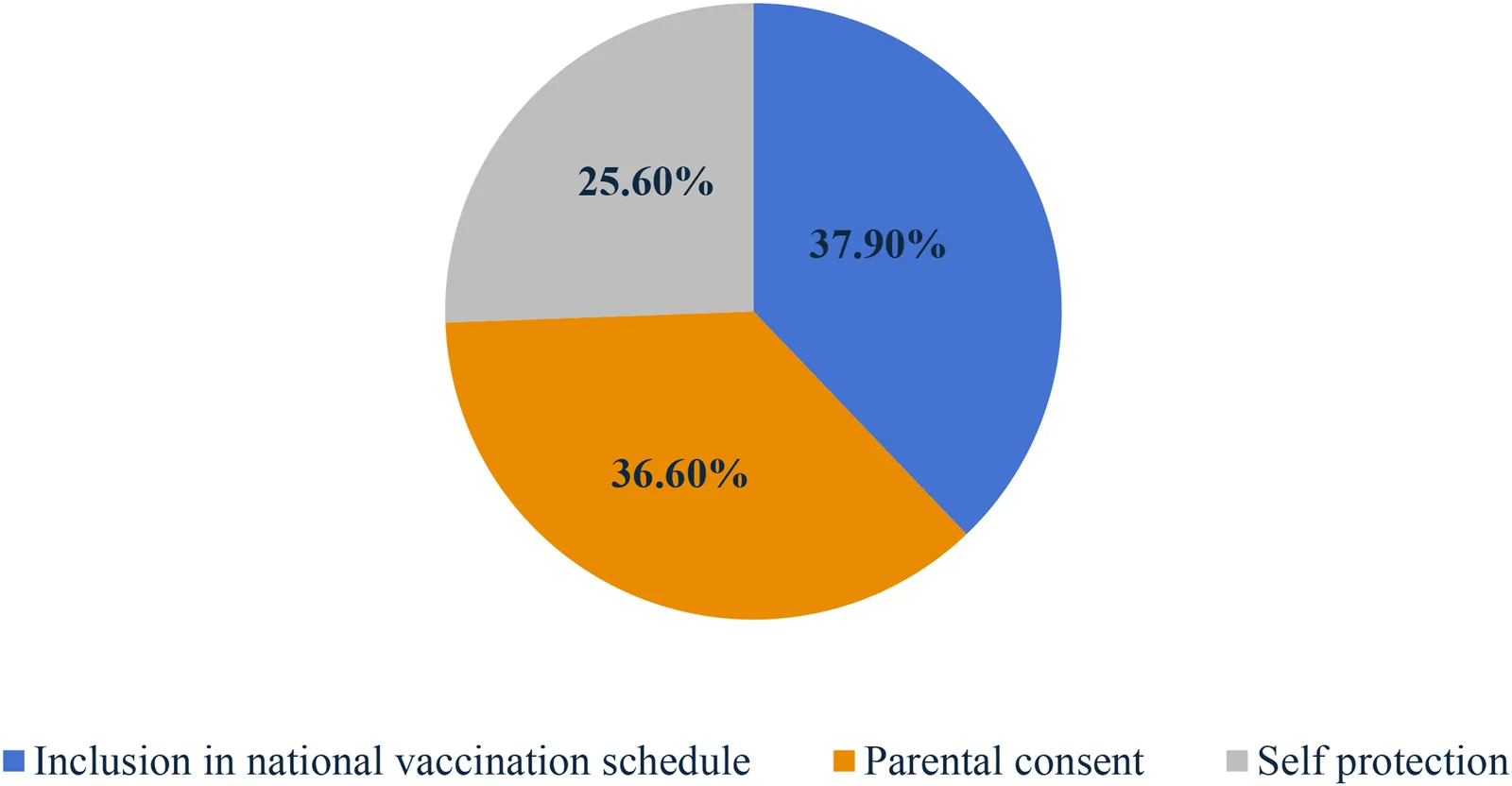
Reasons for receiving the HPV vaccine among vaccinated participants (n = 383).

## Discussion

This study identified significant cultural and structural barriers to HPV vaccination among young women residing in the UAE who participated in the study. Vaccine uptake remained low, with approximately one-quarter of participants reporting vaccination and over one third being uncertain of their vaccination status, complicating the interpretation of vaccination prevalence within the study population. This uncertainty may reflect poor recall or limited awareness, low engagement with the healthcare system, poor communication with healthcare providers, or the young age at which the school-based vaccination has occurred [8,15,16].

Cultural resistance was higher among younger Emirati women and those outside healthcare fields, whereas healthcare affiliation was associated with greater acceptance. Given the convenience-based sampling strategy and demographic composition of the study population, these findings should be interpreted as exploratory rather than nationally representative. Nevertheless, the findings highlight a potential area for targeted education and culturally sensitive public health strategies to improve vaccine uptake in at-risk groups.

### Safety concerns

A prominent barrier identified in this study was fear of vaccine-related side effects, reported by nearly half of non-vaccinated participants, indicating that safety concerns are a primary driver of hesitancy among young women within this study population. Similar concerns have been reported internationally [17–20], although the higher prevalence observed in this study suggests increased anxiety, exposure to misinformation, or less established vaccine safety messaging in the UAE

Despite well-documented vaccine safety [21,22], misconceptions persist, highlighting the need for targeted safety-focused education and reassurance from healthcare providers during routine clinical encounters. However, reducing this hesitancy may require more than repeating international safety messaging. Presumptive recommendation, where providers state the vaccine is due rather than asking if it is wanted, has been shown to increase uptake [23,24], as does motivational interviewing [25,26]. To the best of our knowledge, neither strategy has been evaluated in the context of HPV vaccination in Gulf countries. Future studies assessing the effectiveness of communication approaches in increasing HPV vaccine uptake within this regional context are warranted.

### Sociocultural and family influences

More than one-fourth of participants reported embarrassment when inquiring about the HPV vaccine, and many perceived the topic as culturally taboo, indicating that social norms surrounding sexual health shape vaccination decisions among this study population. Similar barriers, including stigma and cultural sensitivity, have been consistently reported in the UAE and across the region [17,27–30]. Counselling that frames HPV vaccination as routine preventive care (rather than related to sexual activity) should be integrated into adolescent and young adult clinical encounters to reduce stigma and facilitate vaccination.

Family influence was a major determinant of HPV vaccination decisions. Over one-quarter of unvaccinated participants reported fear of judgment from family members, and parental consent was identified as the second most common reason for vaccine uptake, underscoring the continued role of family structures in young women’s health choices in the UAE [31]. Similar patterns have been reported regionally, where parental and spousal support strongly influence vaccine acceptance [18,29,31]. Concurrently, nearly one-third of participants perceived themselves as “too young” for vaccination. This finding may reflect multiple interpretations; some participants may genuinely have been within or near the recommended vaccination age range and had not yet had the opportunity to receive the vaccine. However, it may also reflect the common misperception in conservative societies that premarital abstinence reduces the need for early HPV vaccination [17,28–30]. Regardless of the underlying reason, This finding highlights the need for clearer communication regarding the recommended timing and preventive benefits of HPV vaccination, particularly given that vaccination is most effective prior to sexual exposure [32]. Family-centered educational approaches that emphasize vaccine timing and efficacy may help foster informed and supportive decision-making environments.

### Access, healthcare engagement, and policy

Less than one-third of participants reported receiving information about the HPV vaccine from a healthcare provider, which may reflect limited routine healthcare engagement among younger healthy individuals or reluctance to initiate discussions due to embarrassment. Physician recommendation is consistently identified as one of the strongest predictors of vaccination, and its absence is a major barrier [8,18,20,30,33–39]. However, its impact may be constrained in this age group by infrequent opportunistic healthcare contact. While provider-initiated counselling during routine gynaecology and primary-care encounters remains an important complementary strategy [33,40,41], improving coverage within this group may depend more heavily on accessible education through schools, universities and broader public health campaigns.

Nearly two-thirds of unvaccinated women reported willingness to receive the HPV vaccine if it were offered at their university or workplace, and a similar proportion identified cost as a barrier. These findings align with evidence from school and community-based vaccination programmes demonstrating improved uptake when vaccines are delivered in accessible, familiar settings [30–32,42]. Financial constraints have also been consistently identified as a major barrier to vaccination, including in higher-income contexts [12,13,21,22,43,44]. Taken together, these findings support the implementation of on-site vaccination and awareness programmes in educational and workplace settings, alongside subsidised access, to address both logistical and economic barriers and improve vaccine uptake in this population.

National immunisation policy significantly influenced acceptance, with over one-third of vaccinated participants reporting schedule-based recommendations and nearly two-thirds reporting greater willingness when the HPV vaccine was framed as cancer prevention rather than STI protection. Therefore, policy endorsement and vaccine framing are seemingly key determinants of uptake within this study population. This aligns with MENA-region evidence showing higher coverage in countries with integrated public health programmes [45] and UAE data demonstrating increased parental acceptance following Ministry of Health recommendations [31]. Clear health authority support and cancer-focused public messaging remain essential in culturally sensitive settings [18,30,46], though whether this framing effect holds outside a survey setting has not been tested.

Healthcare affiliation emerged as the strongest predictor of HPV vaccine acceptance, likely due to greater exposure to health education. Although healthcare workers show higher awareness overall [45], persistent knowledge gaps across provider roles highlight the need for ongoing professional education [17,30]. Acceptance was also higher among older women, consistent with increased cumulative health exposure and international trends [45], and among non-Emirati participants, potentially reflecting greater cultural sensitivity surrounding STI-related topics within GCC national populations. These findings suggest potential value in culturally tailored, profession-specific, and community-based educational interventions to address persistent discrepancies in vaccine acceptance.

Geographic variation in cultural resistance was noted, with participants from Abu Dhabi reporting the highest acceptance and lowest hesitation toward HPV vaccination. This likely reflects earlier implementation of school-based vaccination and sustained educational efforts, which have been associated with higher uptake [47]. In contrast, participants from other Emirates expressed greater uncertainty, possibly due to later program adoption and reduced public visibility. These findings suggest that differences in vaccine attitudes may exist across Emirates and warrant further investigation using more representative sampling approaches.

Collectively, these exploratory findings identify several leverage points for targeted policy and clinical interventions. Addressing the compounded barriers identified in this study may require a multilevel approach combining culturally tailored education on vaccine safety and efficacy [15], proactive clinician counselling [8], family engagement [18,31], accessible on-site delivery strategies [32,42], and consistent public health messaging that emphasises cancer prevention [46]. As noted throughout, much of the evidence supporting these individual strategies comes from outside the Gulf, and whether they transfer without adaptation to a setting with stronger family involvement in health decisions and distinct healthcare-seeking patterns remains an open question that further local research should address. If confirmed in broader, representative populations, integrating HPV vaccination into routine gynaecologic care has the potential to strengthen cervical cancer prevention efforts and, hence, contribute to reducing reduce the burden of cervical cancer in the UAE [8].

### Strengths and limitations

This study represents the first large-scale examination in the UAE of cultural and structural barriers to HPV vaccination, providing context-specific evidence to inform future interventions. The study employs a large sample size, which augments the statistical power of the study and reduces potential sampling error. Although most responses were from Abu Dhabi, Dubai and Sharjah, the inclusion of participants from multiple Emirates provides broader representation within the UAE context. Furthermore, the bilingual questionnaire improved accessibility and ensured cultural and linguistic relevance.

Limitations include convenience sampling through social media platforms and university mailing lists, which may have introduced selection bias and overrepresentation of younger, educated, digitally connected, and healthcare worker participants, whose predominance limits the generalisability of the results. The questionnaire was adapted from previously validated tools, but the adapted version was not formally re-validated in this population. Differing interpretation of questionnaire items across health literacy may partly explain the high proportion reporting uncertainty about vaccination status, and reliance on self-reported data introduces selection and recall bias. Given the stigma around this topic, responses may also reflect social desirability bias, likely leading to underestimation of true hesitancy. While anonymous online administration of the questionnaire reduces this bias, it does not eliminate it. These limitations suggest the reported findings should be interpreted as exploratory and hypothesis generating rather than a population level estimates of barrier prevalence or vaccination coverage in the UAE.

Although several demographic differences in HPV Cultural Barriers Scale scores reached statistical significance, effect sizes were generally small, except for healthcare background, which was moderate. This indicates limited practical significance and suggests that the findings may partly reflect the large sample size rather than meaningful population-level differences. Overall, demographic characteristics appear to be weak predictors of cultural barriers, with other factors likely playing a more important role in HPV vaccine uptake.

Future studies should employ representative sampling and longitudinal or qualitative designs to clarify the extent of cultural influences on HPV vaccination. In addition, future work should explore school-based settings as a critical platform for early intervention and vaccination prior to sexual debut to optimize preventive impact.

## Conclusion

The burden on HPV-related diseases continues to represent an important public health concern in many regions, and vaccination uptake remains suboptimal in many regions, including the UAE, despite the availability of effective preventive measures. The findings of this exploratory study suggest that improving HPV vaccine uptake may require coordinated, multi-level strategies. Key priorities include family education, proactive counselling by healthcare providers, and improved access through on-site vaccination in organisational settings. Reducing financial barriers and using culturally appropriate messaging that frames HPV vaccination primarily as cancer prevention are also critical. If confirmed in broader representative populations, integrating these structural, cultural, and informational strategies may enhance vaccination coverage and help reduce the long-term burden of HPV-related disease in the UAE.

## Abbreviations

CI: Confidence Interval
GCC: Gulf Cooperation Council
HPV: Human Papilloma Virus
MENA: Middle East and North Africa
MoHAP: Ministry of Health and Prevention
OR: Odds Ratio
RAK: Ras Al Khaimah
REC: Research Ethics Committee
SD: Standard Deviation
SPSS: Statistical Package for the Social Sciences
STI: Sexually Transmitted Infection
UAE: United Arab Emirates
UAQ: Umm Al Quwain
WHO: World Health Organisation.
