# Supplementary material for: Cultural and structural barriers to HPV vaccine acceptance among young women in the United Arab Emirates
Source: PLoS One. 2026 Oct 30;21(10):e0346507. doi: 10.1371/journal.pone.0346507 (PMC13722194; doi:10.1371/journal.pone.0346507)
Supplement: Supplementary file 1 [file pone.0346507.s001.docx]

Dear Editorial Board,

We would like to thank you and the anonymous reviewers for taking the time to review our paper that was previously titled “Cultural and Structural Barriers to HPV Vaccine Acceptance among Young Women in the United Arab Emirates”, which was submitted to PLOS One Journal. We appreciate your expertise and constructive feedback and hope to address it in an effort to make the paper more comprehensive. Revisions to the manuscript and replies to reviewers’ comments are outlined below.

**Reviewer #1**

Reviewer 1:

OVERALL ASSESSMENT

This manuscript addresses an important and underexplored topic — barriers to HPV vaccination among young women in the UAE — in a setting that is culturally distinctive and underrepresented in the global literature. The sample size is a genuine strength, and the bilingual questionnaire is appropriate for the context. Some of the descriptive findings, particularly around cancer versus STI framing and access facilitation, are practically relevant.

However, the paper has several serious methodological problems that affect the validity and generalisability of its findings. These concern the sampling strategy, the interpretability of the primary outcome variable, the analytical approach, a data availability statement that does not comply with PLOS ONE policy, and a factual reference error. Taken together, they suggest the paper is making claims that substantially exceed what the data can support. I recommend Major Revisions, with the expectation that the authors substantially reframe their claims and address the methodological concerns below.

*Thank you for your thoughtful and constructive feedback. We have substantially revised the manuscript to address concerns regarding sampling, outcome interpretation, analytical approach, data availability compliance, and reference accuracy, and have moderated our claims accordingly.*

| S.no. | Original comments of the reviewer | Reply by the author(s) | Changes done on page number and line number |
| --- | --- | --- | --- |
| 1. | The sampling strategy severely limits generalizability  Participants were recruited via convenience sampling through WhatsApp, Instagram, X, and a university mailing list. The resulting sample is 77.7% non-Emirati, 88.2% single, 76.4% aged 18-24, and 44.8% healthcare students or workers. This is not a representative sample of young women in the UAE — it is a sample of educated, digitally connected, largely expatriate women, a substantial proportion of whom are already health-literate by virtue of their professional training.  This matters because the UAE's population is demographically unusual: the large non-national majority has a very different socioeconomic and cultural profile from the Emirati minority, and both groups are internally heterogeneous. A sample that is nearly four-fifths non-Emirati and nearly half healthcare-affiliated cannot generate population-level estimates of vaccination coverage or barrier prevalence for UAE women as a whole. Yet the paper consistently presents its findings in population-level terms — in the abstract, the discussion, and the conclusions — without adequate qualification.  The authors acknowledge convenience sampling as a limitation but treat it as a minor caveat rather than a fundamental constraint on what can be claimed. The paper must be substantially reframed to present its findings as exploratory and hypothesis-generating, specific to the sample characteristics, and not as representative estimates for UAE women. All population-level language in the abstract, discussion, and conclusion must be revised accordingly. | Thank you for this valuable comment. We agree that the convenience sampling strategy and demographic composition of the study population limit the generalisability of the findings. In response, we revised the manuscript to more clearly present the study as exploratory rather than representative of all women in the UAE. Specifically, population-level wording was replaced with sample-specific language across the abstract, discussion, and conclusion (e.g., referring to “the study sample” rather than “women in the UAE”). We also expanded the limitations section to acknowledge the overrepresentation of younger, non-Emirati, digitally connected, and healthcare-affiliated participants. Additionally, we emphasised the need for future studies using more representative sampling approaches across diverse population groups in the UAE. | Page 1, lines 21-23  Page 2, lines 33-34  Page 2, lines 47-52  Page 16, lines 394-395  Page 16, lines 402-405  Page 17, line 417  Page 17, line 433  Pag 20, line 549  Page 21, lines 573-575  Page 21, line 577  Page 21, line 585  Page 22, lines 611-613  Page 23, line 653  Page 23, line 658 |
| 2. | The vaccination outcome variable is uninterpretable for 38.4% of participants The most important number in the paper may be the finding that 38.4% of participants did not know whether they had been vaccinated against HPV. This is a larger group than either the vaccinated (26.2%) or unvaccinated (35.3%) categories, yet it receives almost no attention in the paper.  This has two serious consequences. First, the reported vaccination rate of 26.2% is uninterpretable as a coverage estimate, since the true rate could be anywhere between 26% and 64% depending on the true vaccination status of the "don't know" group. The paper should not present 26.2% as a coverage figure without explicitly acknowledging this uncertainty and its implications.  Second, the exclusion of the "don't know" group from the primary analysis (the Cultural Barriers Scale and logistic regression, which are conducted only among the 514 self-reported non-vaccinated women) is not justified. The barrier profiles of this group may differ systematically from both vaccinated and unvaccinated participants. Their exclusion introduces potential bias that the authors do not acknowledge. The authors should describe the characteristics and barrier profiles of the "don't know" group, explain their exclusion, and discuss what this means for the validity of the analysis.  Moreover, not knowing one's vaccination status is itself a substantively interesting finding — it may reflect low health system engagement, poor communication from providers, or the age at which school-based vaccination occurred. This deserves explicit discussion rather than being treated as a data management issue. | Thank you for your valuable feedback. In response, we 1) revised the results section to clarify that the reported 26.2% represents self-reported HPV vaccination and that the true vaccination coverage remains uncertain due to the large proportion of participants (38.4%) who were unaware of their vaccination status, as presented in Table 2.  2) We have revised the methods section accordingly. Participants who reported not knowing their vaccination status were no longer excluded from the analyses. Instead, they were retained as a separate category in order to preserve the study sample and better reflect uncertainty regarding HPV vaccination status within the population.  3) We also highlighted the importance of this subgroup in the discussion to address how this uncertainty regarding vaccination status can reflect limited vaccine awareness, gaps in healthcare communication, inadequate documentation, or vaccination during adolescence when healthcare decisions were parent-directed. | 1) Page 9, lines 220-224 2) Page 7, lines 196-209 3) Page 16, lines 398-400 |
| 3. | The logistic regression outcome is not adequately defined  Table 5 presents a multivariable logistic regression of factors associated with "presence of HPV cultural barriers." The paper does not explain how this binary outcome variable was derived from the HPV Cultural Barriers Scale, which is a continuous Likert-based instrument. Was a median split used? A pre-specified threshold? Some other criterion? Without knowing how the binary outcome was constructed, the regression results cannot be interpreted or evaluated. This must be stated explicitly in the methods.  More broadly, dichotomising a continuous scale for logistic regression is analytically suboptimal and loses information. If the authors wish to examine predictors of cultural barrier scores, linear regression on the continuous scale would be more appropriate and more powerful. If a binary outcome is retained, the rationale and cut-point must be justified. | We agree that dichotomising a continuous Likert-based scale may result in loss of information and reduced statistical power. Accordingly, the analysis was revised, and multiple linear regression was conducted using the continuous HPV Cultural Barriers Scale score as the dependent variable instead of logistic regression. The Methods, Results, and Table 5 have been updated accordingly | Page 7, lines 194-209  Page 14, lines 300-308  Table 5 |
| 4. | The analysis is restricted to non-vaccinated women without adequate justification. The Cultural Barriers Scale analysis and the logistic regression are conducted only among the 514 women who reported not being vaccinated. The 383 vaccinated women are excluded from these analyses without explanation. This creates an analytical inconsistency: the paper aims to understand barriers to vaccination but compares barrier profiles only within the non-vaccinated group, missing the opportunity to compare vaccinated and non-vaccinated women directly — which would be the more informative analysis. The authors should either justify the restriction to non-vaccinated women or restructure the analysis to compare vaccinated and non-vaccinated groups, which would provide more meaningful insight into which barriers actually discriminate between the two groups. | We thank the reviewer for this valuable comment. In response, the analysis was revised to include the total study sample (n = 1455), including vaccinated, non-vaccinated, and participants who don’t know their vaccination status. The Methods, Results, and Table 4 and Table 5 have been updated accordingly. | Page 7, lines 196-209  Page 13, lines 294-296  Table 4  Table 5 |
| 5. | Data availability does not comply with PLOS ONE policy  The data availability statement indicates that datasets are available from the corresponding authors on reasonable request. PLOS ONE's data policy explicitly states that this formulation is insufficient. The journal requires that all data underlying the findings be fully available without restriction at the time of publication, either within the manuscript, in Supporting Information files, or deposited in a recognised public repository with a persistent identifier. The authors must either deposit the anonymised dataset in a public repository such as Figshare, Dryad, or Zenodo and provide the DOI, or include it as a Supporting Information file. The current statement does not meet the journal's requirements and must be corrected before the manuscript can be accepted. | We thank the reviewer for highlighting this issue. The data availability statement has been revised to comply with PLOS ONE policy, and the fully anonymised dataset has now been deposited in Zenodo with a corresponding DOI provided in the manuscript. | Page 26, line 728 |
| 6. | Reference error  Reference 4 is cited to support statistics on cervical cancer incidence and mortality (over 660,000 cases and 350,000 deaths in 2022). However, reference 4 (Wéber et al., 2024) is a paper about global bladder cancer mortality — it has no relevance to cervical cancer epidemiology. This must be corrected with an appropriate citation, such as the relevant WHO GLOBOCAN data or the WHO cervical cancer fact sheet. | Thank you for this important observation. We agree that the previously cited reference was not appropriate for supporting cervical cancer incidence and mortality statistics. The reference has now been corrected and replaced with the appropriate WHO source reporting global cervical cancer epidemiology data for 2022. | Page 26, line 734 (reference 1) |
| 7. | The "too young" barrier requires more careful interpretation  Nearly 30% of non-vaccinated participants reported feeling too young for vaccination. The paper interprets this as a misconception reflecting assumptions about premarital abstinence. While this is plausible, an alternative interpretation is that some participants may actually be within or close to the recommended vaccination age range and may not yet have had the opportunity to be vaccinated — in which case this is not a barrier in the behavioural sense but simply a timing issue. The paper should distinguish between these interpretations or acknowledge the ambiguity. | We thank the reviewer for this insightful comment. We agree that perceiving oneself as “too young” for HPV vaccination may reflect multiple interpretations rather than solely representing a misconception related to premarital abstinence. In response, the Discussion section has been revised to acknowledge that some participants may have been within or near the recommended vaccination age range and therefore may not yet have had sufficient opportunity or clinical recommendation to initiate vaccination. The revised text also clarifies that this perception may represent a timing-related consideration rather than a behavioural barrier in some cases. | Page 2; lines 45-47  Page 18; lines 462-467 |
| 8. | Effect sizes in Table 4 are small despite statistical significance  The statistically significant differences in Cultural Barriers Scale scores across demographic groups are accompanied by small effect sizes: Cohen's d values of 0.196 (age), 0.169 (nationality), and 0.356 (healthcare background), and an eta-squared of 0.023 for Emirate of residence. With a sample of 1,455, the study has substantial power to detect trivially small differences. The authors should comment on the practical significance of these differences rather than treating statistical significance as evidence of meaningful variation. The healthcare background finding (Cohen's d = 0.356) is the most practically meaningful, but even this is a modest effect. | We agree with the reviewer and a limitation statement is added to the Discussion to emphasise that, although several between-group differences reached statistical significance, the effect sizes were generally small, likely reflecting the large sample size. This has been further expanded in the Discussion with an additional paragraph to provide clearer interpretation and context. | Page 22, lines 615-618 |
| 9. | Strengths section is overstated  The authors describe the study as "highly significant" and "robust" in the strengths section. These are judgements for readers and reviewers to make, not claims for authors to assert. The strengths section should describe what the study actually did well — the sample size, bilingual instrument, multi-Emirate coverage — without self-congratulatory framing. | Thank you for your comment. We agree that the strengths section should be presented in an objective and descriptive manner. We have therefore revised the section to remove subjective terms such as “highly significant” and “robust,” and to focus instead on the specific methodological strengths of the study, including sample size, bilingual instrument design, and contextual coverage. | Page 21, line 590-600 |
| 10. | Figure 4 citation  Reference 4 in the context of the bladder cancer paper aside, the citation for the WHO cervical cancer burden statistics should be verified against a primary WHO source and updated to reflect the most current available data. | Thank you for this valuable comment. In response, we reviewed and updated the citation supporting the WHO cervical cancer burden statistics to ensure it reflects the most current and appropriate primary source data. | Page 26, line 734 (reference 1) |

SUMMARY
This paper addresses a genuinely important topic in an underexplored setting. Its descriptive findings — particularly around cancer framing, access facilitation, and the role of healthcare affiliation — are useful and relevant. However, the convenience sampling strategy limits generalisability far more than the authors acknowledge; the large proportion of participants with unknown vaccination status fundamentally undermines the coverage estimate and the primary analysis; the regression outcome variable is not adequately defined; and the data availability statement does not meet PLOS ONE's policy requirements. The authors are asked to substantially reframe their claims, address the analytical gaps identified above, and correct the data availability and reference errors before the manuscript can be considered further.

*Thank you for this insightful and balanced evaluation. We have revised the manuscript to acknowledge the limitations of convenience sampling, clarified the outcome variable and handling of unknown vaccination status, strengthened the analytical approach, corrected the data availability statement to meet PLOS ONE requirements, and amended reference errors, with corresponding moderation of our claims.*

**Reviewer #2**

Thank you for your interesting paper.

I have made a few comments below and I hope you find them useful.

***We thank the reviewer for their constructive and helpful comments. We have carefully addressed each point and revised the manuscript accordingly.***

| S.no. | Original comments of the reviewer | Reply by the author(s) | Changes done on page number and line number |
| --- | --- | --- | --- |
| 1. | Line 99: Clarify whether or not males were excluded, as the respondents seemed to be 100% female (line 148). | Thank you for your comment. We confirm that the study population included only female participants. This has now been explicitly clarified in the inclusion criteria section to ensure consistency with the reported sample characteristics. | Page 5, line 124 |
| 2. | Line 100: Clarify how you determined whether or not the questionnaire was complete/ incomplete, and how any responses were deemed inconsistent. | Thank you for this comment. We clarified the criteria used to define incomplete and inconsistent responses in the Methods section. Incomplete questionnaires were defined as surveys with missing mandatory items required for analysis, while inconsistent responses referred to logically contradictory answers across related questions. The revised text has been updated accordingly. | Page 5, lines 126-127 |
| 3. | Line 111: You refer to the validated measures further on, but it would be useful to include them here and briefly outline how these measures were included, i.e. in full (?), in part? | Thank you for this suggestion. We revised the Methods section to clarify that the questionnaire items were adapted from previously validated instruments rather than reproduced in full, and updated our references. We also specified that selected items related to knowledge, barriers, attitudes, and practices were modified to align with the study objectives and the target population. | Page 5, lines 139-151 |
| 4. | Line 116: Did the pilot assess language and/or cultural relevance? | Thank you for this comment. We have clarified that the pilot study was conducted to assess both the linguistic clarity and cultural relevance of the questionnaire items before final distribution. | Page 6, line 156 |
| 5. | Line 131: How were inaccuracies determined? | Thank you for this comment. We clarified that each questionnaire response was reviewed during data screening to identify incomplete submissions, substantial missing data, and logically inconsistent answers across related items (e.g., reporting no HPV awareness but indicating vaccination) before inclusion in the final analysis. We edited it and replaced “inaccuracies” with responses containing “inconsistencies or contraindications” | Page 6, lines 170-171 |
| 6. | Line 161 and 259: With the young demographic, how likely is it that these individuals would regularly engage with the heath service. In your discussion later on, you argue about the importance of physician advice, which is fair enough, but many young people may not attend a physician unless they are ill. I think it may be useful to re-frame the argument in lines 256- 260 unless it is standard practice in your country for everyone to engage regularly with the health service. | We thank the reviewer for this important observation. We agree that routine healthcare engagement among younger individuals is often limited. We have therefore revised the relevant section of the discussion to acknowledge that the influence of physician recommendation, while well established, may be constrained in younger populations due to infrequent healthcare contact. We now place greater emphasis on the need for alternative, more accessible delivery platforms such as university and workplace-based vaccination programmes and broader public health outreach, while still retaining provider-initiated counselling during opportunistic encounters as a complementary strategy. | Page 19, lines 478-486  **(Access, healthcare engagement, and policy** section of the discussion**)** |
| 7. | Line 313: You correctly state that the best time is before the start of sexual activity, so why not include schools here?" | We thank the reviewer for this insightful suggestion. We agree that school-aged populations represent a key target group for HPV vaccination. We revised the relevant section to explicitly include school settings within our future recommendations as important platforms for early intervention and improved vaccine uptake prior to sexual debut. . | Page 23, lines 646-648 |

**Reviewer #3**

Introduction: It is very informative; helps set the stage and discuss the HPV vaccine situation in the Middle East.

*We thank the interviewer for their positive feedback*

| S.no. |  | Reply by the author(s) | Changes done on page number and line number |
| --- | --- | --- | --- |
| 1. | Methodology:   1) Would make sure to clarify inclusion criteria more clearly in line with age requirements etc. 2) Questionnaire development has to be more exact; please specify which articles or literature review were used to develop the questionnaire; HPV vaccine knowledge and practices are well-studied domains internationally; please explain if validated tools were used and if not, why new tools were developed. How were those tools tested or validated? 3) Analysis plan needs more detail and structure 4) Overall, the methodology section can be tightened; some obvious details are discussed at length and others are glanced over | We thank the reviewer for their time and valuable feedback. Based on this:  1) We clarified the inclusion criteria more clearly what the participants were included based on.  2) We revised the methodology to clarify that the questionnaire items were adapted from previously validated instruments and updated our references.  3) We have revised the statistical analysis plan was expanded and rewritten to include more details. We specified the statistical tests used to compare Cultural Barriers Scale scores across participant characteristics (independent-samples t-tests and one-way ANOVA) and clarified the use of multiple linear regression to identify independent predictors of cultural barrier scores.  4) The methodology was reviewed overall and revised and refined. | 1) Page 5, lines 124-128 2) Page 5, lines 139-151 3) Pages 7, lines 188-192 4) Methodology section |
| 2. | Results:   1) For characterstics, the information is split among 4 paragraphs each of which is 2 lines; it can be cleaned up and only the most important information discussed. 2) When talking about cultural barriers, some of the results were surprising but might not have been accurately represented. For example, 58.8% is more than half but might not be considered to be “most”. 3) The results section show a lot of analysis but some of it was not discussed in the methodology section. Please make sure to align the two and put forward a clear analysis plan and then implement it in results 4) Multiple linear regression needs a lot more details and the table (5) leaves more to be desired. 5) Overall, the results section needs to be overhauled dramatically. I do believe there are good results but it would benefit greatly from better presentation and sectioning as well as figures. | We thank the reviewer for their time and valuable feedback. Based on this:  1)We have merged the four short paragraphs in the Knowledge and Uptake subsection into two, focusing on the most relevant findings of the study to improve clarity and readability.  2) We have replaced "most" with the exact percentage (58.8%) in the abstract to provide a more precise representation of the findings. We also reviewed the manuscript for similar language and added a sentence in the Cultural Barriers subsection reporting that only 30.5% of participants knew where to access the vaccine, thereby providing a more balanced representation of awareness and attitudes.  3-4) Statistical analysis was revised, and multiple linear regression was conducted using the continuous HPV Cultural Barriers Scale score as the dependent variable instead of logistic regression. The Methods, Results, and Table 5 have been updated accordingly. We reviewed the Statistical Analysis section in the methods against the analyses reported in Results and identified that age had been omitted from the list of variables compared using independent t-tests, this has been corrected, and a statement specifying that effect sizes (Cohen's d, eta-squared) were calculated has been added.  5) We have restructured the Results with a dedicated subheading for the regression analysis | 1) Page 9, lines 220-226   Page 9, lines 232-235   2) Page 2 line 34   Page 7, lines 182-211 (Statistical analysis section of methodology)   3) Page 9, lines 224-226   Pages 11, lines 260-266  Table 5 |
| 3. | Discussion:   1) The discussion section only superficially discusses the objectives of the study. For example, when talking about safety concerns, there needs to be more discussion and analysis regarding these concerns including actionable points or future research gaps that can be pursued; this applies to a number of other sections where the discussion is superficial although there is a lot of good detailed work published that can help provide some solutions. 2) Additionally, the introduction sets the stage for how the MidEast is unique when it comes to HPV; as such, it may not be justified to apply international solutions to the region without some qualifications or analysis as to whether they would be as effective; it might be prudent to check whether there have been initiatives studied in the region itself and what sort of modifications or approaches they adopted 3) Limitations of the questionnaire and its development need to be discussed and mentioned; more discussion regarding social desirability bias as well 4) Overall, the discussion section needs to synthesize the results more coherently among the already established body of knowledge, trying to clearly highlight how the current issues and barriers align and what can be done to address them. It needs major overhaul. | We thank the reviewer for their comments and valuable feedback.   1) We expanded the Discussion to provide greater interpretation of the findings, identify actionable interventions, and highlight future research priorities. In particular, within the Safety Concerns subsection, we added a paragraph discussing presumptive recommendation and motivational interviewing as evidence-based strategies to address vaccine hesitancy, while noting that neither approach has yet been evaluated for HPV vaccination in Gulf countries. Additional discussion and future research considerations were also incorporated throughout the sociocultural, healthcare engagement, policy, and synthesis sections. 2) We agree that international strategies may not be applicable in our study region. Interventional research regarding this topic is unfortunately scarce in the region. We have added in the synthesis paragraph of the discussion section that the efficacy of the proposed solutions remains untested in the region. 3) We expanded the Limitations section to note that the adapted questionnaire was not re-validated. We also noted that differing interpretation of items across health literacy may partly explain the high proportion of participants reporting uncertainty about their vaccination status**.** Social desirability bias was also added, noting that anonymous online administration reduces, but does not eliminate it. 4) We agree that the discussion section needs to be refined, and have heavily edited that part of the manuscript based on the helpful suggestions of all the esteemed reviewers. We have highlighted the barriers and proposed solutions in each segment of the discussion. The synthesis paragraph was also edited so that the barriers are presented as reinforcing one another, and the recommended multilevel approach now follows directly from that framing | 1) Page 17, lines 419-428 2) Page 20, line 553; Page 21, lines 581-585 3) Page 22, lines 605-611 4) Discussion |
| 4. | Conclusion:   1) Would add a line about background in the conclusion | Thank you for this comment. We have added a brief line at the beginning of the conclusion regarding HPV-related diseases and its burden as a background and updating the section to better reflect the study findings and their implications. | Page 23, lines 651-653 |
| 5. | Figures: Figure 1 can be improved; using percentages can help; additionally, the bars can be split to highlight important demographics if deemed useful; that can make the figure a lot more useful | We thank you very much for this valuable suggestion. Figure 1 has been revised to include percentages alongside frequencies for improved interpretability. In addition, the revised figure now presents all surveyed HPV vaccination-related factors, including both barriers and facilitators to vaccine uptake, providing a more comprehensive overview of influences on vaccination decisions among non-vaccinated women. While demographic stratification was considered, the purpose of this figure was to provide an overall summary of barriers and facilitators in the study population. | Figure 1  Page 12 |
