# Supplementary material for: Cultural and structural barriers to HPV vaccine acceptance among young women in the United Arab Emirates
Source: PLoS One. 2026 Oct 30;21(10):e0346507. doi: 10.1371/journal.pone.0346507 (PMC13722194; doi:10.1371/journal.pone.0346507)
Supplement: Peer review history

# Peer review: Decision Letter 0

**DOI:** 10.1371/journal.pone.0346507.r001

**Authors:** Omar Enzo Santangelo

18 Jun 2026

PLOS One

Dear Dr. Kelly,

Thank you for submitting your manuscript to PLOS ONE. After careful consideration, we feel that it has merit but does not fully meet PLOS ONE’s publication criteria as it currently stands. Therefore, we invite you to submit a revised version of the manuscript that addresses the points raised during the review process.

==============================

Dear Authors,

the manuscript requires major revisions. Please respond to the reviewers' requests point by point.

==============================

Please submit your revised manuscript by Aug 02 2026 11:59PM. If you will need more time than this to complete your revisions, please reply to this message or contact the journal office at plosone@plos.org. When you're ready to submit your revision, log on to https://www.editorialmanager.com/pone/ and select the 'Submissions Needing Revision' folder to locate your manuscript file.

- A letter that responds to each point raised by the academic editor and reviewer(s). You should upload this letter as a separate file labeled 'Response to Reviewers'.
- A marked-up copy of your manuscript that highlights changes made to the original version. You should upload this as a separate file labeled 'Revised Manuscript with Track Changes'.
- An unmarked version of your revised paper without tracked changes. You should upload this as a separate file labeled 'Manuscript'.

If you would like to make changes to your financial disclosure, please include your updated statement in your cover letter. Guidelines for resubmitting your figure files are available below the reviewer comments at the end of this letter.

If applicable, we recommend that you deposit your laboratory protocols in protocols.io to enhance the reproducibility of your results. Protocols.io assigns your protocol its own identifier (DOI) so that it can be cited independently in the future. For instructions see: https://journals.plos.org/plosone/s/submission-guidelines#loc-laboratory-protocols. Additionally, PLOS ONE offers an option for publishing peer-reviewed Lab Protocol articles, which describe protocols hosted on protocols.io. Read more information on sharing protocols at https://plos.org/protocols?utm_medium=editorial-email&utm_source=authorletters&utm_campaign=protocols.

As the corresponding author, your ORCID iD is verified in the submission system and will appear in the published article. PLOS supports the use of ORCID, and we encourage all coauthors to register for an ORCID iD and use it as well. Please encourage your coauthors to verify their ORCID iD within the submission system before final acceptance, as unverified ORCID iDs will not appear in the published article. *Only*  the individual author can complete the verification step; PLOS staff *cannot*  verify ORCID iDs on behalf of authors.

We look forward to receiving your revised manuscript.

Kind regards,

Omar Enzo Santangelo

Academic Editor

PLOS One

**Journal Requirements:**

1. When submitting your revision, we need you to address these additional requirements.

Please ensure that your manuscript meets PLOS ONE's style requirements, including those for file naming. The PLOS ONE style templates can be found at

https://journals.plos.org/plosone/s/file?id=wjVg/PLOSOne_formatting_sample_main_body.pdf and

https://journals.plos.org/plosone/s/file?id=ba62/PLOSOne_formatting_sample_title_authors_affiliations.pdf

2. In the online submission form, you indicated that “The datasets used and/or analysed during the current study are available from the corresponding authors on reasonable request.”

All PLOS journals now require all data underlying the findings described in their manuscript to be freely available to other researchers, either a. In a public repository, b. Within the manuscript itself, or c. Uploaded as supplementary information.

This policy applies to all data except where public deposition would breach compliance with the protocol approved by your research ethics board. If your data cannot be made publicly available for ethical or legal reasons (e.g., public availability would compromise patient privacy), please explain your reasons on resubmission and your exemption request will be escalated for approval.

3. Your ethics statement should only appear in the Methods section of your manuscript. If your ethics statement is written in any section besides the Methods, please move it to the Methods section and delete it from any other section. Please ensure that your ethics statement is included in your manuscript, as the ethics statement entered into the online submission form will not be published alongside your manuscript.

4. Please include captions for your Supporting Information files at the end of your manuscript, and update any in-text citations to match accordingly. Please see our Supporting Information guidelines for more information: http://journals.plos.org/plosone/s/supporting-information.

5. If the reviewer comments include a recommendation to cite specific previously published works, please review and evaluate these publications to determine whether they are relevant and should be cited. There is no requirement to cite these works unless the editor has indicated otherwise.

[Note: HTML markup is below. Please do not edit.]

Reviewers' comments:

**Comments to the Author**

1. Is the manuscript technically sound, and do the data support the conclusions?

Reviewer #1: No

Reviewer #2: Yes

Reviewer #3: Yes

**********

2. Has the statistical analysis been performed appropriately and rigorously?

Reviewer #1: No

Reviewer #2: Yes

Reviewer #3: No

**********

3. Have the authors made all data underlying the findings in their manuscript fully available?

The PLOS Data policy

Reviewer #1: No

Reviewer #2: Yes

Reviewer #3: Yes

**********

4. Is the manuscript presented in an intelligible fashion and written in standard English?

Reviewer #1: Yes

Reviewer #2: Yes

Reviewer #3: Yes

**********

**Reviewer #1:**  PEER REVIEW COMMENTS

Manuscript: PONE-D-26-10357

Cultural and Structural Barriers to HPV Vaccine Acceptance among Young Women in the United Arab Emirates

---

OVERALL ASSESSMENT

This manuscript addresses an important and underexplored topic — barriers to HPV vaccination among young women in the UAE — in a setting that is culturally distinctive and underrepresented in the global literature. The sample size is a genuine strength, and the bilingual questionnaire is appropriate for the context. Some of the descriptive findings, particularly around cancer versus STI framing and access facilitation, are practically relevant.

However, the paper has several serious methodological problems that affect the validity and generalisability of its findings. These concern the sampling strategy, the interpretability of the primary outcome variable, the analytical approach, a data availability statement that does not comply with PLOS ONE policy, and a factual reference error. Taken together, they suggest the paper is making claims that substantially exceed what the data can support. I recommend Major Revisions, with the expectation that the authors substantially reframe their claims and address the methodological concerns below.

---

MAJOR COMMENTS

1. The sampling strategy severely limits generalisability

Participants were recruited via convenience sampling through WhatsApp, Instagram, X, and a university mailing list. The resulting sample is 77.7% non-Emirati, 88.2% single, 76.4% aged 18-24, and 44.8% healthcare students or workers. This is not a representative sample of young women in the UAE — it is a sample of educated, digitally connected, largely expatriate women, a substantial proportion of whom are already health-literate by virtue of their professional training.

This matters because the UAE's population is demographically unusual: the large non-national majority has a very different socioeconomic and cultural profile from the Emirati minority, and both groups are internally heterogeneous. A sample that is nearly four-fifths non-Emirati and nearly half healthcare-affiliated cannot generate population-level estimates of vaccination coverage or barrier prevalence for UAE women as a whole. Yet the paper consistently presents its findings in population-level terms — in the abstract, the discussion, and the conclusions — without adequate qualification.

The authors acknowledge convenience sampling as a limitation but treat it as a minor caveat rather than a fundamental constraint on what can be claimed. The paper must be substantially reframed to present its findings as exploratory and hypothesis-generating, specific to the sample characteristics, and not as representative estimates for UAE women. All population-level language in the abstract, discussion, and conclusion must be revised accordingly.

2. The vaccination outcome variable is uninterpretable for 38.4% of participants

The most important number in the paper may be the finding that 38.4% of participants did not know whether they had been vaccinated against HPV. This is a larger group than either the vaccinated (26.2%) or unvaccinated (35.3%) categories, yet it receives almost no attention in the paper.

This has two serious consequences. First, the reported vaccination rate of 26.2% is uninterpretable as a coverage estimate, since the true rate could be anywhere between 26% and 64% depending on the true vaccination status of the "don't know" group. The paper should not present 26.2% as a coverage figure without explicitly acknowledging this uncertainty and its implications.

Second, the exclusion of the "don't know" group from the primary analysis (the Cultural Barriers Scale and logistic regression, which are conducted only among the 514 self-reported non-vaccinated women) is not justified. The barrier profiles of this group may differ systematically from both vaccinated and unvaccinated participants. Their exclusion introduces potential bias that the authors do not acknowledge. The authors should describe the characteristics and barrier profiles of the "don't know" group, explain their exclusion, and discuss what this means for the validity of the analysis.

Moreover, not knowing one's vaccination status is itself a substantively interesting finding — it may reflect low health system engagement, poor communication from providers, or the age at which school-based vaccination occurred. This deserves explicit discussion rather than being treated as a data management issue.

3. The logistic regression outcome is not adequately defined

Table 5 presents a multivariable logistic regression of factors associated with "presence of HPV cultural barriers." The paper does not explain how this binary outcome variable was derived from the HPV Cultural Barriers Scale, which is a continuous Likert-based instrument. Was a median split used? A pre-specified threshold? Some other criterion? Without knowing how the binary outcome was constructed, the regression results cannot be interpreted or evaluated. This must be stated explicitly in the methods.

More broadly, dichotomising a continuous scale for logistic regression is analytically suboptimal and loses information. If the authors wish to examine predictors of cultural barrier scores, linear regression on the continuous scale would be more appropriate and more powerful. If a binary outcome is retained, the rationale and cut-point must be justified.

4. The analysis is restricted to non-vaccinated women without adequate justification

The Cultural Barriers Scale analysis and the logistic regression are conducted only among the 514 women who reported not being vaccinated. The 383 vaccinated women are excluded from these analyses without explanation. This creates an analytical inconsistency: the paper aims to understand barriers to vaccination, but compares barrier profiles only within the non-vaccinated group, missing the opportunity to compare vaccinated and non-vaccinated women directly — which would be the more informative analysis. The authors should either justify the restriction to non-vaccinated women or restructure the analysis to compare vaccinated and non-vaccinated groups, which would provide more meaningful insight into which barriers actually discriminate between the two groups.

5. Data availability does not comply with PLOS ONE policy

The data availability statement indicates that datasets are available from the corresponding authors on reasonable request. PLOS ONE's data policy explicitly states that this formulation is insufficient. The journal requires that all data underlying the findings be fully available without restriction at the time of publication, either within the manuscript, in Supporting Information files, or deposited in a recognised public repository with a persistent identifier. The authors must either deposit the anonymised dataset in a public repository such as Figshare, Dryad, or Zenodo and provide the DOI, or include it as a Supporting Information file. The current statement does not meet the journal's requirements and must be corrected before the manuscript can be accepted.

---

MINOR COMMENTS

6. Reference error

Reference 4 is cited to support statistics on cervical cancer incidence and mortality (over 660,000 cases and 350,000 deaths in 2022). However, reference 4 (Wéber et al., 2024) is a paper about global bladder cancer mortality — it has no relevance to cervical cancer epidemiology. This must be corrected with an appropriate citation, such as the relevant WHO GLOBOCAN data or the WHO cervical cancer fact sheet.

7. The "too young" barrier requires more careful interpretation

Nearly 30% of non-vaccinated participants reported feeling too young for vaccination. The paper interprets this as a misconception reflecting assumptions about premarital abstinence. While this is plausible, an alternative interpretation is that some participants may actually be within or close to the recommended vaccination age range and may not yet have had the opportunity to be vaccinated — in which case this is not a barrier in the behavioural sense but simply a timing issue. The paper should distinguish between these interpretations or acknowledge the ambiguity.

8. Effect sizes in Table 4 are small despite statistical significance

The statistically significant differences in Cultural Barriers Scale scores across demographic groups are accompanied by small effect sizes: Cohen's d values of 0.196 (age), 0.169 (nationality), and 0.356 (healthcare background), and an eta-squared of 0.023 for Emirate of residence. With a sample of 1,455, the study has substantial power to detect trivially small differences. The authors should comment on the practical significance of these differences rather than treating statistical significance as evidence of meaningful variation. The healthcare background finding (Cohen's d = 0.356) is the most practically meaningful, but even this is a modest effect.

9. Strengths section is overstated

The authors describe the study as "highly significant" and "robust" in the strengths section. These are judgements for readers and reviewers to make, not claims for authors to assert. The strengths section should describe what the study actually did well — the sample size, bilingual instrument, multi-Emirate coverage — without self-congratulatory framing.

10. Figure 4 citation

Reference 4 in the context of the bladder cancer paper aside, the citation for the WHO cervical cancer burden statistics should be verified against a primary WHO source and updated to reflect the most current available data.

---

SUMMARY

This paper addresses a genuinely important topic in an underexplored setting. Its descriptive findings — particularly around cancer framing, access facilitation, and the role of healthcare affiliation — are useful and relevant. However, the convenience sampling strategy limits generalisability far more than the authors acknowledge; the large proportion of participants with unknown vaccination status fundamentally undermines the coverage estimate and the primary analysis; the regression outcome variable is not adequately defined; and the data availability statement does not meet PLOS ONE's policy requirements. The authors are asked to substantially reframe their claims, address the analytical gaps identified above, and correct the data availability and reference errors before the manuscript can be considered further.

**Reviewer #2:**  Thank you for your interesting paper.

I have made a few comments below and I hope you find them useful.

Line 99: Clarify whether or not males were excluded, as the respondents seemed to be 100% female (line 148).

Line 100: Clarify how you determined whether or not the questionnaire was complete/ incomplete, and how any responses were deemed inconsistent.

Line 111: You refer to the validated measures further on, but it would be useful to include them here and briefly outline how these measures were included, i.e. in full (?), in part?

Line 116: Did the pilot assess language and/or cultural relevance?

Line 131: How were inaccuracies determined?

Line 161 and 259: With the young demographic, how likely is it that these individuals would regularly engage with the heath service. In your discussion later on, you argue about the importance of physician advice, which is fair enough, but many young people may not attend a physician unless they are ill. I think it may be useful to re-frame the argument in lines 256- 260 unless in it is standard practice in your country for everyone to engage regularly with the health service.

Line 313: You correctly state that the best time is before the start of sexual activity, so why not include schools here?

**Reviewer #3:**  ----- Introduction:

- It is very informative; helps set the stage and discuss the HPV vaccine situation in the Middle East.

----- Methodology:

- Would make sure to clarify inclusion criteria more clearly in line with age requirements etc.

- Questionnaire development has to be more exact; please specify which articles or literature review were used to develop the questionnaire; HPV vaccine knowledge and practices are well-studied domains internationally; please explain if validated tools were used and if not, why new tools were developed. How were those tools tested or validated?

- Analysis plan needs more detail and structure

- Overall, the methodology section can be tightened; some obvious details are discussed at length and others are glanced over

---- Results

- For characterstics, the information is split among 4 paragraphs each of which is 2 lines; it can be cleaned up and only the most important information discussed

- When talking about cultural barriers, some of the results were surprising but might not have been accurately represented. For example, 58.8% is more than half but might not be considered to be “most”.

- The results section show a lot of analysis but some of it was not discussed in the methodology section. Please make sure to align the two and put forward a clear analysis plan and then implement it in results

- Multiple linear regression needs a lot more details and the table (5) leaves more to be desired.

- Overall, the results section needs to be overhauled dramatically. I do believe there are good results but it would benefit greatly from better presentation and sectioning as well as figures.

---- Discussion

- The discussion section only superficially discusses the objectives of the study. For example, when talking about safety concerns, there needs to be more discussion and analysis regarding these concerns including actionable points or future research gaps that can be pursued; this applies to a number of other sections where the discussion is superficial although there is a lot of good detailed work published that can help provide some solutions.

- Additionally, the introduction sets the stage for how the MidEast is unique when it comes to HPV; as such, it may not be justified to apply international solutions to the region without some qualifications or analysis as to whether they would be as effective; it might be prudent to check whether there have been initiatives studied in the region itself and what sort of modifications or approaches they adopted

- Limitations of the questionnaire and its development need to be discussed and mentioned; more discussion regarding social desirability bias as well

- Overall, the discussion section needs to synthesize the results more coherently among the already established body of knowledge, trying to clearly highlight how the current issues and barriers align and what can be done to address them. It needs major overhaul.

---- Conclusion: Would add a line about background in the conclusion

Figures:

- Figure 1 can be improved; using percentages can help; additionally, the bars can be split to highlight important demographics if deemed useful (for example, knowledge strata; HCW, etc); that can make the figure a lot more useful

-

**********

what does this mean?). If published, this will include your full peer review and any attached files.

If you choose “no”, your identity will remain anonymous but your review may still be made public.

**Do you want your identity to be public for this peer review?**  For information about this choice, including consent withdrawal, please see our Privacy Policy

Reviewer #1: No

Reviewer #2: No

Reviewer #3: **Yes:**  KAMEL SAMARA

**********

[NOTE: If reviewer comments were submitted as an attachment file, they will be attached to this email and accessible via the submission site. Please log into your account, locate the manuscript record, and check for the action link "View Attachments". If this link does not appear, there are no attachment files.]

To ensure your figures meet our technical requirements, please review our figure guidelines: https://journals.plos.org/plosone/s/figures

You may also use PLOS’s free figure tool, NAAS, to help you prepare publication quality figures: https://journals.plos.org/plosone/s/figures#loc-tools-for-figure-preparation.

NAAS will assess whether your figures meet our technical requirements by comparing each figure against our figure specifications.

# Author comment: Author response to Decision Letter 1

**DOI:** 10.1371/journal.pone.0346507.r002

1 Sep 2026

Dear Editorial Board,

We would like to thank you and the anonymous reviewers for taking the time to review our paper that was previously titled “Cultural and Structural Barriers to HPV Vaccine Acceptance among Young Women in the United Arab Emirates”, which was submitted to PLOS One Journal. We appreciate your expertise and constructive feedback and hope to address it in an effort to make the paper more comprehensive. Revisions to the manuscript and replies to reviewers’ comments are outlined below.

Attachment Submitted filename: Response to Reviewers.docx

# Peer review: Decision Letter 1

**DOI:** 10.1371/journal.pone.0346507.r003

**Authors:** Omar Enzo Santangelo

12 Oct 2026

Cultural and Structural Barriers to HPV Vaccine Acceptance among Young Women in the United Arab Emirates.

PONE-D-26-10357R1

Dear Dr. Kelly,

We’re pleased to inform you that your manuscript has been judged scientifically suitable for publication and will be formally accepted for publication once it meets all outstanding technical requirements.

Within one week, you’ll receive an e-mail detailing the required amendments. When these have been addressed, you’ll receive a formal acceptance letter and your manuscript will be scheduled for publication.

An invoice will be generated when your article is formally accepted. Please note, if your institution has a publishing partnership with PLOS and your article meets the relevant criteria, all or part of your publication costs will be covered. Please make sure your user information is up-to-date by logging into Editorial Manager at Editorial Manager® and clicking the ‘Update My Information' link at the top of the page. For questions related to billing, please contact billing support.

If your institution or institutions have a press office, please notify them about your upcoming paper to help maximize its impact. If they’ll be preparing press materials, please inform our press team as soon as possible -- no later than 48 hours after receiving the formal acceptance. Your manuscript will remain under strict press embargo until 2 pm Eastern Time on the date of publication. For more information, please contact onepress@plos.org.

Kind regards,

Omar Enzo Santangelo

Academic Editor

PLOS One

Additional Editor Comments (optional):

Reviewers' comments:

Reviewer's Responses to Questions

**Comments to the Author**

Reviewer #2: All comments have been addressed

Reviewer #4: All comments have been addressed

**********

2. Is the manuscript technically sound, and do the data support the conclusions?

Reviewer #2: Yes

Reviewer #4: Yes

**********

3. Has the statistical analysis been performed appropriately and rigorously?

Reviewer #2: Yes

Reviewer #4: Yes

**********

4. Have the authors made all data underlying the findings in their manuscript fully available?

The PLOS Data policy

Reviewer #2: Yes

Reviewer #4: Yes

**********

5. Is the manuscript presented in an intelligible fashion and written in standard English?

Reviewer #2: Yes

Reviewer #4: Yes

**********

Reviewer #2: Thank you for addressing my comments and I wish you success in your ongoing research in this interesting area of research.

Reviewer #4: (No Response)

**********

what does this mean?). If published, this will include your full peer review and any attached files.

If you choose “no”, your identity will remain anonymous but your review may still be made public.

**Do you want your identity to be public for this peer review?**  For information about this choice, including consent withdrawal, please see our Privacy Policy

Reviewer #2: No

Reviewer #4: No

**********

# Editor report: Acceptance letter

**DOI:** 10.1371/journal.pone.0346507.r004

**Authors:** Omar Enzo Santangelo

PONE-D-26-10357R1

PLOS One

Dear Dr. Kelly,

I'm pleased to inform you that your manuscript has been deemed suitable for publication in PLOS One. Congratulations! Your manuscript is now being handed over to our production team.

At this stage, our production department will prepare your paper for publication. This includes ensuring the following:

* All references, tables, and figures are properly cited

* All relevant supporting information is included in the manuscript submission,

* There are no issues that prevent the paper from being properly typeset

You will receive further instructions from the production team, including instructions on how to review your proof when it is ready. Please keep in mind that we are working through a large volume of accepted articles, so please give us a few days to review your paper and let you know the next and final steps.

Lastly, if your institution or institutions have a press office, please let them know about your upcoming paper now to help maximize its impact. If they'll be preparing press materials, please inform our press team within the next 48 hours. Your manuscript will remain under strict press embargo until 2 pm Eastern Time on the date of publication. For more information, please contact onepress@plos.org.

You will receive an invoice from PLOS for your publication fee after your manuscript has reached the completed accept phase. If you receive an email requesting payment before acceptance or for any other service, this may be a phishing scheme. Learn how to identify phishing emails and protect your accounts at https://explore.plos.org/phishing.

If we can help with anything else, please email us at customercare@plos.org.

Thank you for submitting your work to PLOS One and supporting open access.

Kind regards,

PLOS One Editorial Office Staff

on behalf of

Dr. Omar Enzo Santangelo

Academic Editor

PLOS One
